# Effects of hormone replacement therapy on lens opacity, serum inflammatory cytokines, and antioxidant levels

**DOI:** 10.1080/07853890.2021.1928275

**Published:** 2021-05-22

**Authors:** Donghyun Jee, Sang Hee Park, Ho Sik Hwang, Hyun Seung Kim, Man Soo Kim, Eun Chul Kim

**Affiliations:** a Department of Ophthalmology, St. Vincent Hospital, The Catholic University of Korea, Seoul, Korea; b Institute of Clinical Medicine Research, College of Medicine, The Catholic University of Korea, Seoul, Korea

**Keywords:** Hormone replacement therapy (HRT), lens opacity, Scheimpflug imaging system, inflammatory cytokines, antioxidants

## Abstract

**Purpose:**

To evaluate the effect of hormone replacement therapy (HRT) on lens opacity as measured by Scheimpflug densitometry, serum inflammatory cytokines, and antioxidant levels.

**Methods:**

A total of 264 women who were at least five years beyond menopause were included. The control group of 128 patients (Group 1) did not use HRT at any time after menopause. The treatment group of 136 patients (Group 2) used HRT for5 years or more after menopause. Cortical, nuclear, and posterior subcapsular density; pentacam nucleus staging (PNS); pentacam densitometry of zone (PDZ) as measured using a Scheimpflug imaging system (Pentacam); and antioxidant and inflammatory cytokines activities in serum using multiplex bead analysiswere examined.

**Results:**

Uncorrected visual acuity (logMAR) of group1 was significantly worse than those of group 2 (*p* < .05). Group1 was significantly more myopic than group 2 (*p* < .05). Nuclear and posterior subcapsular density, PNS, and percentage of PDZ in group 1 were significantly higher than those of group 2 (*p* < .05). The serum IL-1m. IL-6, IL-8, and TGF-, concentrations of group 1 were significantly higher than those of group 2, and the serum catalase, superoxide dismutase 1 (SOD 1), and superoxide dismutase 2 (SOD 2) fluorescence intensities of group 1 were significantly lower than those of group 2 (*p* < .05).

**Conclusions:**

Long-term use of HRT may have a protective effect against cataract formation. HRT seems to be effective in decreasing inflammation and increasing antioxidant contents in the serum of postmenopausal women.

**KEY MESSAGES**
- Hormone replacement therapy (HRT) decrease lens opacity in postmenopausal women as measured by Scheimpflug densitometry.
- HRT decrease serum inflammatory cytokines and increase antioxidant levelsin the serum of postmenopausal women.
- Long-term use of HRT may have a protective effect against cataract formationin postmenopausal women.

## Introduction

Women have been reported to have worse vision than men of the same age. Several studies have reported that postmenopausal women have a higher prevalence of cataracts than men of a similar age [1,2]. Although the relationship between gender and cataract prevalence is unclear, and there is no clear mechanism for the difference, female sex hormones may play an important role in protecting against cataract progression [2].

Oestrogen and progesterone receptor mRNAs are present in the eyes, especially in the lens [3]. Oestrogen replacement therapy has some protective effect against apoptosis of lens epithelial cells [4]. However, apoptosis does not seem to play an important role in the occurrence of cataracts because only limited apoptosis was detected in the lens epithelium of cataract patients [5]. Hormone replacement therapy (HRT) has been found to have a modest protective effect on nuclear and posterior subcapsular lens opacities in postmenopausal women [6]. In a study on users of tamoxifen, an antiestrogen drug used for breast cancer, cataract formation was roughly 4as roughly 4hlytamoxifen users compared to a non-user, and the annual cataract rate of tamoxifen users was 6.8% during the follow-up time of 5 years [7].

17-r Oestradiol (E2) could regulate the expression and secretion of inflammatory cytokines (decrease the expression of TNF-es and regulate immune responses [8]. HRT might also have a protective effect against oxidative damage in postmenopausal women [9]. Indeed, systemic inflammatory reaction may play a key role in cataract development [10], and oxidative stress is one of the major risk factors for cataract formation; the activated antioxidant systems may have a protective effect against cataract formation [11].

However, other studies have reported no observed effect of HRT on cataract prevention [12–14]. Other studies have reported the opposite relationship: long periods of HRT use could increase cataract extraction in postmenopausal women, especially those drinking more than one alcoholic drink daily [15].

However, no studies have addressed the effects of HRT on cataract formation using Scheimpflug imaging combined with measurements of serum anti-inflammatory cytokine and antioxidant levels in postmenopausal women. This is the first study to compare cataract densities in different regions of the lens by means of these measurements, comparing postmenopausal women who have been treated with HRT to a control group.

## Methods

This study was a parallel-group, retrospective cohort study. The study was conducted in compliance with Institutional Review Board regulations, sponsor and investigator obligations, and the Declaration of Helsinki. The Institutional Review Board (IRB)/Ethics Committee of Bucheon St. Mary Hospital approved this study protocol (HC13RISI0026).

### Patients

Eligible patients were divided into two groups. The control group (Group 1) consisted of 128 postmenopausal women who were at least 5 years into the postmenopausal period and had never used HRT after menopause. The treatment group (Group 2) consisted of 136 postmenopausal women who had continuously used HRT for at least 5 years starting from the onset of menopause; this group included subjects who received oestrogen and progesterone combination therapy.

This study was conducted at theBucheon St. Mary Hospital from July 2014 to March 2017.

The main inclusion criteria were women who were at least 5 years after menopause, and perimenopausal and early postmenopausal women without contraindications who were experiencing troublesome vasomotor symptoms. The exclusion criteria were: (1) a history of any ocular injury or disorder, infection, inflammation, surgery within the prior 6 months; and (2) any uncontrolled systemic disease such as diabetes mellitus, autoimmune diseases, or significant illness.

### Scheimpflug densitometry

The lens density of each patientom right eye was measured using the Scheimpflug system. Patients were given 1% tropicamide for pupil dilatation and were examined with an Oculus Pentacam (Oculus Inc., Germany). Theutomatic release mode was used to reduce operator-dependent variables. The rotating Scheimpflug camera captures up to 50 slit images of the anterior segment. The Pentacam Scheimpflug densitometric method was used to measure nuclear density, using Pentacam software (Pentacam lens density program; PLDP) and Pentacam nucleus staging (PNS). The average Pentacam densitometry of zone was measured. Lens density at specific area (cortex, nucleus, and posterior subcapsular) was measured at the 3-dimensional representation of the lens image.

### Multiplex bead analysis

Inflammatory cytokines and antioxidants were examined with immunoassay panels (Millipore MILLIPLEX Human Cytokine/Chemokine Panel I Premixed 42 Plex [MPXHCYTO60KPMX42] and Millipore MILLIPLEX Human Oxidative Stress Panel Premixed 5 Plex [H0XSTMAG-18K]; Millipore, Billerica, MA) using a magnetic bead-based immunoassay kit (Luminex 200; Luminex Corp., Austin, TX), according to a previously reportedmethod [16]. Table 1 lists the quantities of inflammatory cytokines and antioxidants found. Serum was incubated with antibody-coated capture beads overnight at 4 °C [16]. Washed beads were further incubated with biotin-labeled anti-human cytokine antibodies and then subjected to streptavidinephycoerythrin incubation. The standard curves of known concentrations of recombinant human cytokines were used to convert fluorescence units to concentrations (pg/mL) [15]. To calculate molecular concentrations in serum, we analysed the median fluorescent intensity data using a 5-parameter logistic or spline curve-fitting method.

**Table 1.** Inflammatory cytokines and antioxidants for serum quantification of patients with menopause.

Human Cytokine Human Cytokine/ Chemokine Panel I	EGF, FGF-2, TGF-β, G-CSF, IFNγ, IL-1β, IL-6, IL-8, IL-12(p40), IL-17A, MCP-1/CCL2/MCAF, MIP-1α, TNFα, VEGF
Human Oxidative Stress Panel	Catalase, SOD1, SOD2, PRX2 (PRDX2), TRX1

### Statistical analysis

Statistical analysis was performed using SPSS software (version 16.0, SPSS Inc., Chicago, IL). The Mann–Whitney *U* test was used for pairwise comparisons of treatment group categorical variables. Outcomes of the continuous variables were analysed using unpaired *t-*tests. A two-sided test with *p* < .05 was considered significant.

## Results

The study was performed from July 2014 to March 2017. The following outcomes were found to be not statistically significant between the groups: age, years from menopause, body mass index (BMI), lifestyle (smoking), concomitant diseases (diabetes mellitus, hypertension), best corrected visual acuity (logMAR), corneal thickness, anterior chamber depth, and lens diameter(*p* > .05).

However, the uncorrected visual acuity (logMAR) of group1 was significantly worse than that of group 2. The mean spherical equivalent refraction (D) of group1 was significantly more myopic than that of group 2 (*p* < .05, Table 2).

**Table 2.** Characteristics of patients with menopause.

	Group 1: HRT(−)	Group 2: HRT(+)
Total patients (Eyes)	128 (128)	136 (136)
Age	56.77 ± 5.97	59.75 ± 5.90
Years from menopause	6.52 ± 1.23	6.23 ± 1.52
Body mass index (BMI)	25.91 ± 3.24	26.13 ± 4.01
Smoker	2 (1.6%)	3 (2.2%)
Diabetes	36 (28.1%)	40 (29.4%)
Hypertension	42 (32.8%)	45 (33.1%)
Uncorrected visual acuity (logMAR)	0.27 ± 0.14	0.10 ± 0.09
Best corrected visual acuity (logMAR)	0.052 ± 0.005	0.056 ± 0.007
Mean spherical equivalent refraction (D)	−0.73 ± 0.68	−0.54 ± 0.47
Corneal thickness	557.54 ± 47.56	545.79 ± 50.70
Anterior chamber depth	2550.25 ± 301.56	2553.75 ± 302.98
Lens diameter	2200.26 ± 821.40	2223.50 ± 651.14

Data represent mean ± standard deviation.

HRT(−): without hormone replacement therapy, HRT(+): with hormone replacement therapy.

Uncorrected visual acuity (logMAR) of group1 was significantly worse than that of group 2. Mean spherical equivalent refraction (D) of group1 was significantly more myopic than that of group 2. (*p* < 0.05, unpaired *t*-test).

### Lens densitometry

The following outcomes were found to be significantly higher for group 1 (no HRT group) compared to group 2 (HRT group): nuclear density (*p* = .04), posterior subcapsular density (*p* = .03), PNS grade (*p* = .01), and average PDZ (%) (*p* = .01). There was no significant difference in cortical density between the two groups (*p* = .37) (Table 3).

**Table 3.** Lens densitometry of each group with menopause.

	Group 1: HRT(−)	Group 2: HRT(+)
Total patients (Eyes)	128 (128)	136 (136)
*Nuclear density	17.17 ± 6.35	15.61 ± 4.38
Cortical density	12.93 ± 4.18	12.89 ± 3.58
*Posterior subcapsular density	8.11 ± 1.79	7.17 ± 1.03
*PNS Grade	1.25 ± 0.46	0.81 ± 0.49
*average PDZ (%)	12.23 ± 1.80	10.02 ± 0.71

Data represent mean ± standard deviation.

HRT(−): without hormone replacement therapy, HRT(+): with hormone replacement therapy.

PNS: Pentacam nucleus staging; PDZ: Pentacam densitometry of zone.

*: *p* < 0.05.

**Figure 1.**
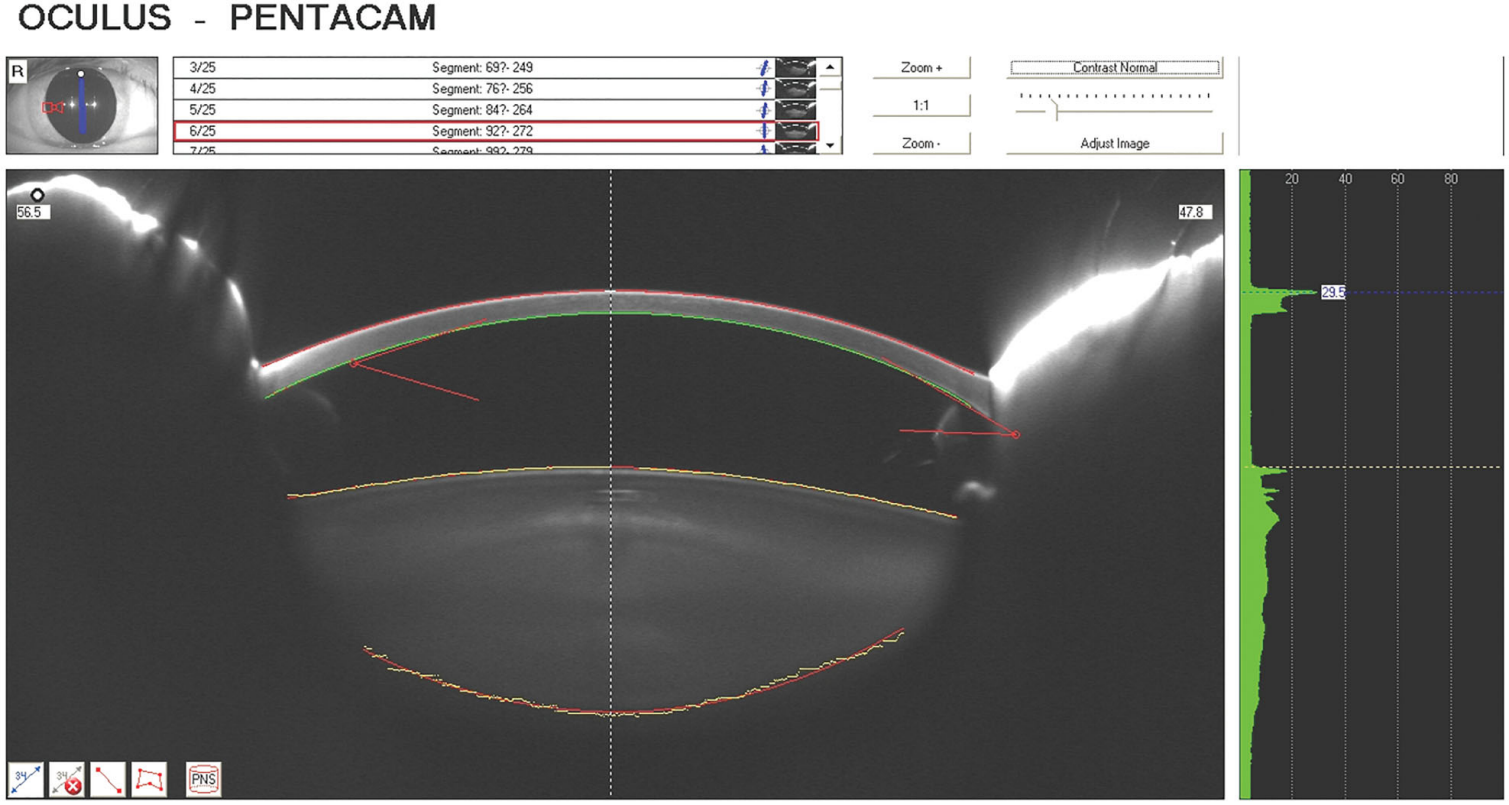
Scheimpflug image of lens densitometry. Numbers indicate the region of lens densitometry (1: cortex, 2: nucleus, 3: posterior subcapsular region).

Figure 1 indicates each part of the lens density measured by the Scheimpflug imaging system.

### Serum oestradiol and progesterone levels

Baseline values for serum oestradiol (8.7 ± 1.23 and 9.1 ± 1.52 pg/ml) and progesterone (65.7 ± 10.76, 68.3 ± 9.95 pg/ml) were comparable between groups 1 and 2. In year 5, serum oestradiol and progesterone (17.5 ± 2.25, 98.6 ± 12.38 pg/ml) of group 2 were significantly greater than those ofgroup 1 (8.9 ± 1.36, 63.8 ± 9.58 pg/ml), respectively (*p* < .05). The year 5/baseline ratio of serum oestradiol and progesterone (1.92, 1.44) of group 2 were also statistically increased compared to group 1 (1.02, 0.97), respectively (*p* < .05) (Table 4).

**Table 4.** Serum oestradiol and progesterone levels.

	Group 1: HRT(−)	Group 2: HRT(+)
Total patients (Eyes)	128 (128)	136 (136)
Baseline oestradiol (pg/ml)	8.7 ± 1.23	9.1 ± 1.52
*Year 5 oestradiol (pg/ml)	8.9 ± 1.36	17.5 ± 2.25
*Oestradiol Ratio (Year 5/baseline)	1.02	1.92
Baseline progesterone (pg/ml)	65.7 ± 10.76	68.3 ± 9.95
*Year 5 progesterone (pg/ml)	63.8 ± 9.58	98.6 ± 12.38
*Progesterone Ratio (Year 5/baseline)	0.97	1.44

Data represent mean ± standard deviation.

HRT(−): without hormone replacement therapy; HRT(+): with hormone replacement therapy.

*: *p* < .05.

### Inflammatory cytokines

**Figure 2.**
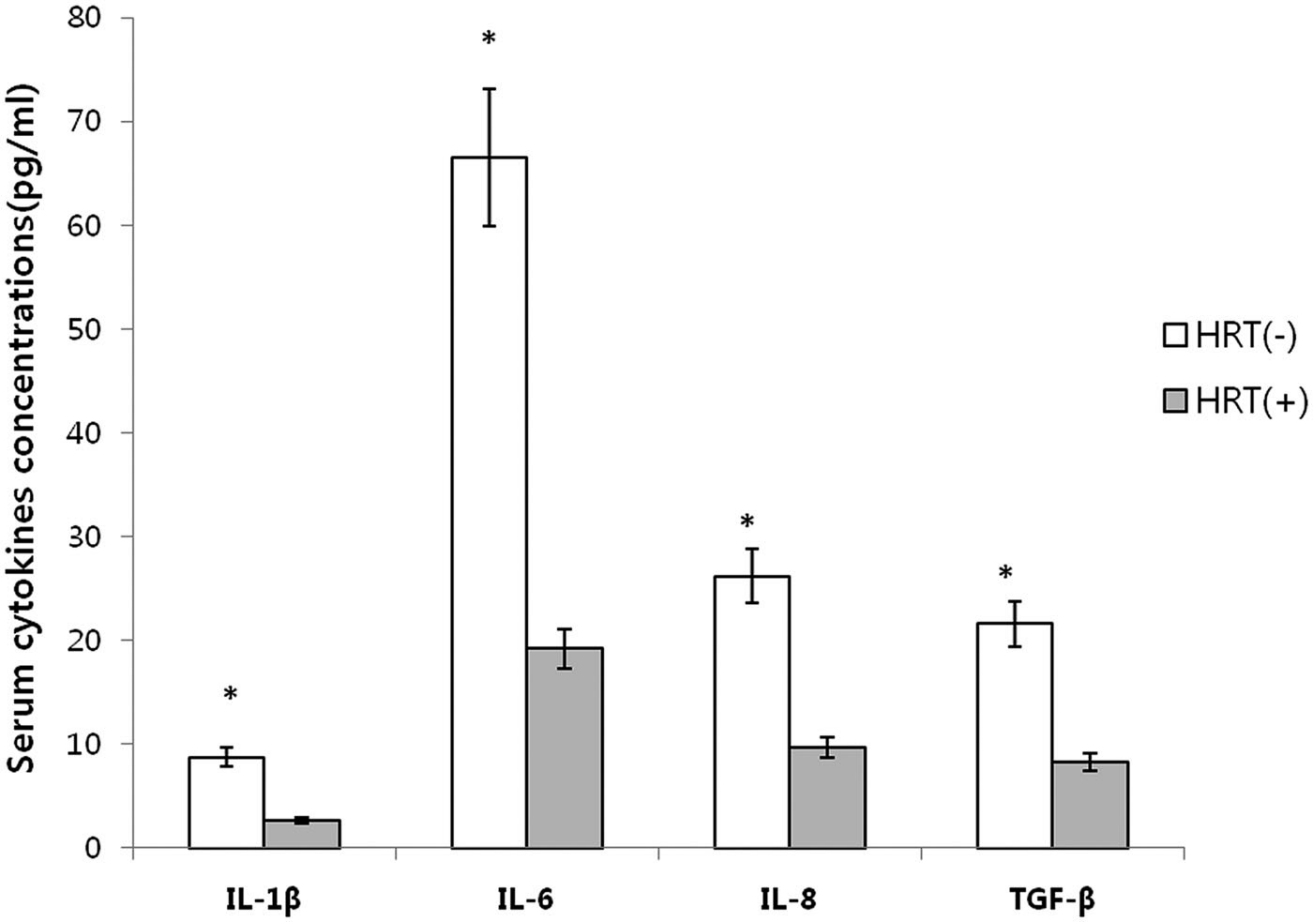
Serum concentrations of inflammatory cytokines. Mean value ± standard deviation. HRT(−): no hormone replacement treatment group, HRT(+): hormone replacement treatment group. Serum concentrations of IL-1βL IL-6, IL-8, and TGF-βof group 1 were statistically higher than those of group 2 (*: *p* < 0.05 by unpaired *t* test).

Serum IL-1β (8.77 ± 1.03, 2.71 ± 0.78 pg/ml, *p* = .03), IL-6 (66.5 ± 8.57, 19.2 ± 2.36 pg/ml, *p* = .01), IL-8 (26.2 ± 3.14, 9.7 ± 1.59 pg/ml, *p* = .02), and TGF-β(21.6 ± 2.79, 8.3 ± 1.37 pg/ml, *p* = .03) concentrations of group 1 were significantly higher than those of group 2, respectively (Figure 2).

### Antioxidants

**Figure 3.**
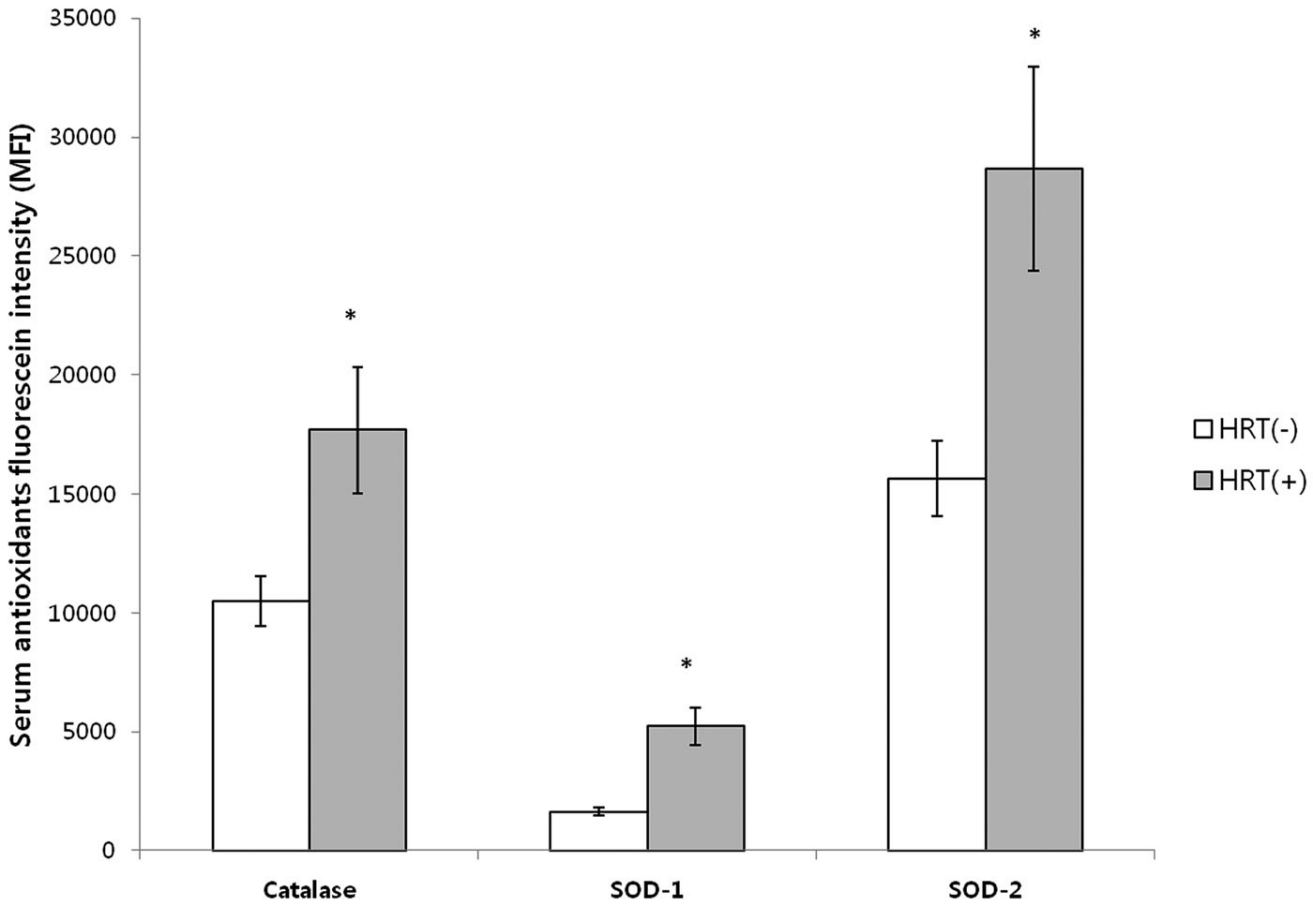
Serum fluorescence intensity of antioxidants. Mean value ± standard deviation. MFI: Mean fluorescence intensity. HRT(−): no hormone replacement treatment group, HRT(+): hormone replacement treatment group. Serum fluorescence intensities of catalase, superoxide dismutase 1 (SOD 1), and superoxide dismutase 2 (SOD 2) of group 1 were statistically lower than those of group 2 (*: *p* < 0.05 by unpaired *t* test).

Serum catalase (10,492.33 ± 886.78, 17,686.5 ± 1354.43 mean fluorescence intensity[MFI], *p* = .03), superoxide dismutase 1 (SOD 1) (1634.57 ± 583.26, 5226.79 ± 1076.59 MFI, *p* = .03),and superoxide dismutase 2 (SOD 2)(15,636.48 ± 2383.67, 28,653.81 ± 4286.54 MFI, *p* = .01) fluorescence intensity of group 1 were significantly lower than those of group 2, respectively (Figure 3).

## Discussion

There are several established methods of cataract density evaluation. The standard classification system based on slitlampbiomicrosopy is the Lens Opacity Classification III (LOCS III) [17]. LOCS III is cost-effective, but it is a subjective method and is influenced by slitlamp settings such as illumination amount and the examinerti level of training [18]. A new objective method, double-pass technology, measures optical aberrations and ocular scatter resulting from the loss of ocular transparency [16]. Another objective classification system of cataract density is Scheimpflug imaging, in which a rotating Scheimpflug camera makes a 3-dimensional image of the crystalline lens by capturing images in different meridians [19].

Both the LOCS III and Scheimpflug imaging measurements have been reported to be correlated with total ultrasound power, cumulative dissipated energy (CDE), and fluid used [18]. However, the Scheimpflug system has a stronger correlation with these phacoemulsification parameters than the LOCS III system [18]. The objective scatter index (OSI), based on the double-pass system, was also correlated with lens density measured by means of Scheimpflug imaging, subjective lens grading, and CDE [16].

Lens density measured by means ofScheimpflug imaging was assessed using the PNS. A negative correlation between lens density and best-corrected visual acuity and a positive correlation between density and total dissipated phacoenergy in the Scheimpflug imaging systems were reported [20]. Thus, the Scheimpflug optical densitometry can be used for both prediction and monitoring of the cataract state [20].

Oestrogen and progesterone receptor mRNAs are widely expressed in the human eye, including the lens [3]. E2 helped in preventing the deteriorating effect of H_2_O_2_ and inhibited cell death, apoptosis and depolymerisation of cytoskeletal proteins in lens epithelial cells (LECs) [21].

Several reports have observed putative protective effects of oestrogen use on the lenses of postmenopausalwomen [22–24]. HRT has also been reported to have a protective association with lens opacity in postmenopausal women [6], including against nuclear and posterior subcapsular opacities [24].

However, some authors have reported that HRT increases cataract development in postmenopausal women [12–14,25]. Therefore, we evaluated the effect of HRT on lens opacity, using Scheimpflug densitometry and measurements of serum inflammatory cytokine levels and antioxidant levels.

In this study, the following outcomes were significantly higher for group 1 compared to group 2: nuclear density (*p* = .04), posterior subcapsular density (*p* = .03), PNS grade (*p* = .01), and average PDZ (%) (*p* = .01) of group 1 (no HRT group). There was also no significant difference in cortical density between the two groups (*p* = .37).

Therefore, the uncorrected visual acuity (logMAR) of group1 was significantly worse than that of group 2 (*p* < .05). We hypothesised that significant higher nuclear density in group 1 resulted in significantly more myopic refraction in group 1 compared group 2 (*p* < .05) (Table 2).

Our main question was whether the expression of inflammatory factors and antioxidant levels can be correlated to the use of hormonal treatment and the incidence of cataracts.

Menopause was reported to increase inflammatory cytokine expression in the cervical mucosa with MIG, MIP-3aa IL-13a IL-6, IL-8, IP-10, and MCP-1 [26]. Ovariectomy (surgical menopause) increased inflammatory markers, TGF-er and oxidative damage in a rat-basedmodel [27]. TGF-dm is involved in the pathophysiology of atopic cataracts [28]. Inflammatory response by IL-1b and possibly IL-6 may play a role in UVR-B-induced cataracts [10].

Oxidative stress was increased after surgical oestrogen deprivation, but mRNA expression of superoxide dismutase (SOD) and glutathione peroxidase (GSH-Px) were resolved after oestrogen replacement therapy [29]. A significant positive relationship was observed between the E2 and reduced glutathione (GSH), SOD, and GSH-Px [29]. The maturity of cataract was associated with significant imbalances between aqueous humour oxidants and antioxidants in terms of decreased SOD, total proteins, and conjugated dienes (CD), as well as increased lipofuscin-like fluorescent endproducts (LLF) [30].

In this study, The year 5 concentration and year 5/baseline ratio of serum oestradiol and progesterone of the HRT group were statistically significantly increased compared to the control group, respectively (*p* < .05) (Table 4). Therefore, HRT increases serum oestradiol and progesterone levels in postmenopausal women compared to control.

We hypothesise that increased inflammatory cytokines and oxidative damage because of depletion of oestrogen can generate cataracts in postmenopausal women.17pausale[men replacement therapy attenuates homocysteine-induced oxidative stress and inflammatory response *via* the PI3-K/Akt signal transduction pathway [31].

In this study, serum IL-1βL IL-6, IL-8, and TGF-, concentrations of group 1 were significantly higher than those of group 2; serum catalase,SOD 1, and SOD 2 fluorescence intensities of group 1 were significantly lower than those of group 2 (*p* < .05).

Therefore, increased expression of inflammatory factors and oxidative stress can generate cataracts in postmenopausal women, and hormone replacement therapy can decrease cataract incidence by attenuating inflammatory factors and oxidative stress.

Kanthan et al. reported that the oral contraceptive pill had a mild protective effect against incident cortical cataracts. However, HRT did not have a significant relationship with the incidence of any type of cataract or cataract surgery [32]. However, in the present study, HRT with oestrogen and progesterone combination therapy had a protective effect on nuclear and posterior subcapsular opacities, but did not have any on cortical-type cataracts (Table 3).

Other studies have reported that long periods of HRT use could increase cataract extraction in postmenopausal women, especially for those drinking more than one alcoholic drink daily. The possible protective effect of oestrogen might be related to endogenous oestrogen. Exogenous oestrogen in form of HRT is not to be regarded as a physiological substitution and could have other effects on the lens [15]. And heavy drinking has been linked to poor nutrition status of antioxidant vitamins A and E, which may have a protective effect against cataract progression [33].

Greater vitamin A intakes might be inversely associated with risk for cataract. Antioxidant vitamin A could be used for postmenopausal women without HRT after menopause [34].

The polyol pathway, also called the sorbitol–aldose reductase (AR) pathway, has been implicated in the development of diabetic complications such as cataracts [35]. Genistein is a naturally occurring compound that structurally belongs to a class of compounds known as isoflavones. It is described as an angiogenesis inhibitor and a phytoestrogen. Genistein inhibits aldose reductase activity and high glucose-induced TGF-os expression in human lens epithelial cells [35]. Therefore, HRT may be a potential therapy for preventing and treating complications associated with diabetes mellitus, such as diabetic cataracts.

In conclusion, for the first time we report that postmenopausal women using HRT for at least 5 years have lower densities of nuclear and posterior subcapsular lens than those who have not used HRT, as measured by using Scheimpflug imaging systems. We also report that postmenopausal women using HRT have lower concentrations of inflammatory cytokines and higher serum fluorescence intensities of antioxidants than those not using HRT.

Based on the studyxi findings, there is room for future research. The measurement of inflammatory cytokines in aqueous humour should be performed in patients who undergo cataract surgery. Indeed, a large multicenter trial with prolonged follow-up for a large number of study participants is needed to conclusively determine the efficacy of HRT on these outcomes. In this study, multivariate analysis was not performed because this study was designed as a case-control study and adopted restricted selection. In the future, multivariate analysis among HRT, cataract density, and inflammatory cytokines and antioxidants levels should be performed.

In conclusion, long-term use of HRT, defined as five years in this study, may have a protective effect against nuclear and posterior subcapsular cataract formation in postmenopausal women. Indeed, HRT may decrease inflammatory response and increase serum antioxidants in postmenopausal women.

## Data Availability

The datasets used and/or analysed during the current study available fromthe corresponding author on reasonable request.
